# The hypothesis of ultraconserved enhancer dispensability overturned

**DOI:** 10.1186/s13059-018-1433-1

**Published:** 2018-05-08

**Authors:** Laura Elnitski, Ivan Ovcharenko

**Affiliations:** 1 Translational and Functional Genomics Branch, National Human Genome Research Institute, National Institutes of Health, Bethesda, MD 20892 USA; 2 Computational Biology Branch, National Center for Biotechnology Information, National Library of Medicine, National Institutes of Health, Bethesda, MD 20892 USA

## Abstract

Two recent studies explore how redundant enhancers in mice really are.

Major advancements in science are grounded in a healthy dose of skepticism. Prior to 2002, during the sequencing phase of the Mouse Genome Project, the discovery of elements harboring unexpectedly high sequence identity to the human genome was interpreted, at first skeptically, as contamination of human DNA in the mouse samples. When contamination was later disproven, ultraconserved elements (UCEs) were formally recognized as 481 genomic segments at least 200 base pairs in length that are perfectly conserved (with no insertions or deletions) between orthologous regions of the human, mouse, and rat genomes [1]. Importantly, due to their frequency, depth of conservation and association with essential developmental genes, UCEs were inferred as being essential for the ontogeny of vertebrate as well as invertebrate species. Despite the enthusiasm, expectations took another step backward, and again skepticism reigned, after the deletion of four of these elements, in 2007, showed no obvious phenotype in E11.5 mouse embryos or adult mice [2]. This finding has perplexed the genomics community for the last decade.

In the meantime, UCEs have been shown to have many functional roles in the genome by acting as enhancers impacting neurological functions and limb development, autoregulatory splicing domains in genes encoding RNA-binding proteins, as contributors to coding exons of genes, or recognition sequences for homeodomain proteins. Moreover, indicative of a diverse functional spectrum, some UCEs are transcribed as non-coding RNAs and upregulated in cancers. Several prior reports also suggested that mutations in UCEs might be associated with neurodevelopmental and immune system disorders. Of utmost consideration, sequence conservation, the strongest indicator of selective pressure in mammalian genomes, shows that non-coding UCEs are under stronger selective pressure than protein-coding genes. Abundant non-human primate mutations in UCEs suggest that UCEs are not mutation-free regions, but rather are regions evolving under extreme negative selection constraint [3]. How could it be possible that these conspicuously conserved regions were phenotypically neutral upon deletion?

Functionally, non-coding UCEs are important because they show enrichment near many developmental genes and are known to drive expression patterns similar to the expression of their flanking genes. Thus, several hypotheses have been proposed to explain the conundrum of dispensable function in the deletion models: (1) redundancy of enhancer function might provide phenotypic stability in mammalian development; (2) precise excision of a compartmentalized genomic structure may have allowed the developing organism to bypass the necessity for it; (3) UCEs might contribute extremely specialized functions which are not measureable in a laboratory environment, but whose loss would decrease fitness of species detrimentally during evolution.

In the January 2018 issue of *Cell*, Dickel et al. [4] have shown, to much anticipation, that the deletion of an UCE leads to a measurable phenotype, despite viability of the enhancer knockout animals (Fig. 1a, b). By deleting UCEs near the essential neuronal transcription factor *Arx* using the CRISPR-Cas9 technique, the team found that mice carrying single or pairwise deletions in nearly all cases showed neurological or growth abnormalities. In support of these findings, mutations in *Arx* cause a variety of severe neurological phenotypes in X-linked disorders, and these four UCEs show enhancer activity in the developing forebrain that is similar to *Arx* gene expression patterns. Notably, previous studies suggested redundant expression patterning from several of the UCEs in this region [5].

**Fig. 1:**
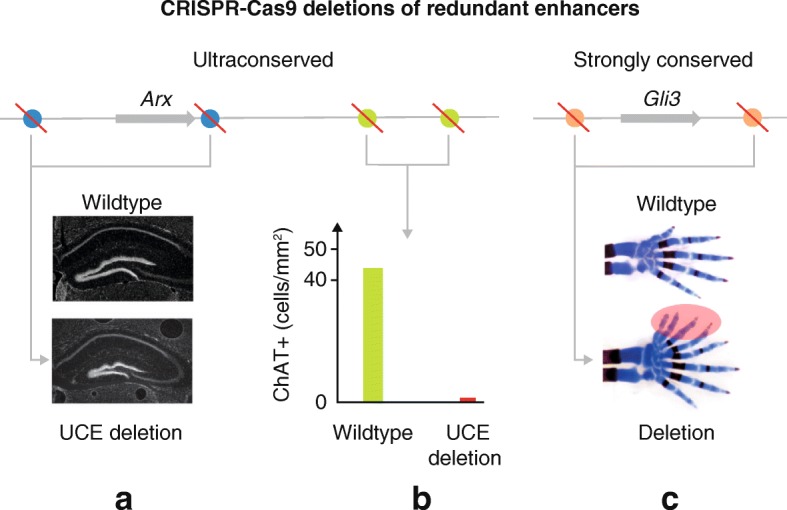
Pairwise deletion of redundant ultraconserved elements in the locus of the mouse *Arx* gene [4] (**a**, **b**) and redundant strongly conserved enhancers of the mouse *Gli3* gene [8] (**c**). A combined deletion of two dorsal forebrain enhancers hs122 and hs123 leads to a smaller dentate gyrus (*white staining*) with disorganized appearance (**a**). A combined deletion of two ventral forebrain enhancers hs119 and hs121 leads to a drastic decrease in the density of striatal cholinergic neuron density (**b**). A combined deletion of two *Gli3* limb enhancers in a sensitized genetic background leads to a severe polydactyly (**c**). *ChAT* choline acetyltransferase, *UCE* ultraconserved element

In findings of this research group headed by Drs Pennacchio, Dickel and Visel, individual deletion of any of four ultraconserved forebrain enhancers near *Arx* produced mice that are both viable and fertile, including hemizygous null males and homozygous null females. Mice missing pairs of the ultraconserved enhancers (to account for potential redundancy) were also viable and fertile. Thus, their results demonstrated that an individual organism’s viability or fertility was not dependent on these, some of the longest UCEs in the human genome. Interpreted another way, the extreme sequence conservation might indicate important biological function, but not developmental essentiality of these sequences.

Upon closer inspection of the phenotypical findings, RNA-seq performed on whole forebrain tissue from male E11.5 embryos, hemizygous null for either single or pairs of enhancers, showed that the double enhancer deletions significantly reduced *Arx* expression, while the expression of all other genes within a large 10 Mb window surrounding these UCEs was largely unaffected. Furthermore, some of the single and pairwise deletions displayed up to 15% reduction in body mass during the developmental time period. Neuroanatomical changes were also reported in some of the deletions including a 62% reduction in choline acetyltransferase expressing neurons in postnatal mouse brain and abnormalities to the anatomical structure of the hippocampus. These findings are consistent with another study of impairment caused by loss of UCE function, as demonstrated by Nolte et al. [6], in which deletion of an UCE associated with limb formation produced viable mice with no gross limb malformations, yet these mice were significantly smaller than controls. The neurodevelopmental impact of UCE sequence changes and their detrimental impact on the long-term fitness of the species are further confirmed by a recent study, in which single nucleotide mutations in *Arx* UCEs were linked to intellectual disability in humans [7].

In the February 2018 issue of *Nature*, another research study performed by the Pennacchio, Dickel and Visel groups showed that redundancy of enhancers, newly observed in UCEs, is widespread among developmental enhancers in mammals [8]. In that study, Osterwalder and colleagues focused their attention on pairwise redundant evolutionary conserved limb enhancers in the loci of the mouse *Gli3* and *Shox2* genes, which are critical for proper limb development (Fig. 1c). CRISPR-Cas9 deletion of pairs of redundant limb enhancers, but not single enhancers, resulted in a phenotypic change in limb skeletal morphology, including polydactyly and variable femur length. To examine the interplay between gene dosage, heterozygous genotypes and redundant enhancer function, the group profiled the effects of pairwise enhancer deletions in a sensitized genetic background carrying heterozygous target gene deletions. The effect of single and pairwise redundant enhancer deletions was amplified in sensitized experiments, suggesting a biological essentiality of redundant enhancers in genetically compromised species. To demonstrate a pronounced impact of these findings relative to the regulatory architectures of an average gene in a mammalian genome, the authors focused on the abundance of redundant enhancers in individual gene loci. They found more than 1000 genes having five or more enhancers carrying redundant activity patterns, which regulate developmental expression in brain, limb and heart tissues. They conclude that enhancer redundancy provides protection against loss of individual regulatory functions and speculate that disease-associated phenotypes may be likely to emerge from gain-of-function enhancer mutations that expand enhancer activities or reposition enhancers relative to their target genes.

Together, these results support the idea that loss of UCEs and other strongly evolutionarily conserved elements can cause defects that may have profound consequences for reproductive success in the wild, but are nevertheless subtle in laboratory mice, due to redundancy of enhancer functions. These losses have been strongly selected against over extremely long evolutionary timescales of hundreds of million years. In contrast, the hemizygous loss of function of the ARX transcription factor does have catastrophic consequences. Male *Arx* gene knockout mice (hemizygous null) die within 2 days after birth and display severe developmental defects, including anomalies in testes and pancreas as well as smaller brains. Moreover, loss-of-function mutations in humans correspond to a series of X-linked disorders including agenesis of the corpus callosum with abnormal genitalia, and syndromic and nonspecific X-linked mental retardation ([9] and references within). This comparison, although indirect, suggests that the tissue-specific loss of enhancer function and its associated reduction of gene expression may have a localized effect on cellular function, which can be tolerated given the presence of normal expression in other cell types; whereas a systemic reduction of the protein product itself cannot be overcome.

In light of these findings, it is interesting to note the recent report of Chen et al. [10], describing imaging studies in 14 pairs of monozygotic twins with attention deficit hyperactivity disorder. Despite a lack of causal gene mutations, affected twins had a significantly smaller right striatum and thalamus, and a trend toward a larger cerebellum, but did not differ in cerebral cortical volume. Affected twins also showed significant differences in DNA methylation patterns associated with some enhancer regions of genes expressed in the altered brain regions. Taken together, these reports offer the possibility that subtle effects, such as loss or alteration of enhancer elements in the genome may be associated with discrete neuroanatomical anomalies. Thus, the long-awaited outcomes of UCE deletion phenotypes may herald a new era in our understanding of complex diseases of the human brain.

## Abbreviation

UCE: Ultraconserved element
